# Observational study of lipid profile and LDL particle size in patients with metabolic syndrome

**DOI:** 10.1186/1476-511X-10-162

**Published:** 2011-09-21

**Authors:** Natalia Sancho-Rodríguez, Francisco V Avilés-Plaza, Esteban Granero-Fernández, Antonio M Hernández-Martínez, María Dolores Albaladejo-Otón, Pedro Martínez-Mernández, Soledad Parra-Pallarés

**Affiliations:** 1 Department of Clinical Analysis, University Hospital Virgen de la Arrixaca, Murcia, Spain; 2 Espinardo Health Center, Murcia, Spain; 3 Endocrinology and Nutrition Services, University Hospital Virgen de la Arrixaca, Murcia, Spain; 4 Department of Clinical Analysis, University Hospital Santa Lucía, Cartagena, Spain

**Keywords:** Atherosclerosis, LDLC particle size, Metabolic Syndrome

## Abstract

**Background:**

The atherogenic lipoprotein phenotype is characterized by an increase in plasma triglycerides, a decrease in high-density lipoprotein cholesterol (HDLc), and the prevalence of small, dense-low density lipoprotein cholesterol (LDLc) particles. The aim of this study was to establish the importance of LDL particle size measurement by gender in a group of patients with Metabolic Syndrome (MS) attending at a Cardiovascular Risk Unit in Primary Care and their classification into phenotypes.

**Subjects and methods:**

One hundred eighty-five patients (93 men and 92 women) from several areas in the South of Spain, for a period of one year in a health centre were studied. Laboratory parameters included plasma lipids, lipoproteins, low-density lipoprotein size and several atherogenic rates were determinated.

**Results:**

We found differences by gender between anthropometric parameters, blood pressure and glucose measures by MS status. Lipid profile was different in our two study groups, and gender differences in these parameters within each group were also remarkable, in HDLc and Apo A-I values. According to LDL particle size, we found males had smaller size than females, and patients with MS had also smaller than those without MS. We observed inverse relationship between LDL particle size and triglycerides in patients with and without MS, and the same relationship between all atherogenic rates in non-MS patients. When we considered our population in two classes of phenotypes, lipid profile was worse in phenotype B.

**Conclusion:**

In conclusion, we consider worthy the measurement of LDL particle size due to its relationship with lipid profile and cardiovascular risk.

## 1. Background

Atherosclerosis and its relevant vascular events including cardiovascular disease (CVD), stroke and peripheral arterial disease (PAD) have become a leading cause of disability and mortality in modern society [1].

A lifestyle summarized as a lack of physical activity and moderate-to-high intake calories seems to be one of the most important causes of rapidly increasing prevalence of the metabolic syndrome (MS) [2].

The indicence of MS is rising and is recognized as a major global health problem. Furthermore, recent guidelines for primary and secondary prevention of coronary artery disease consider MS as a major risk factor for CVD [3]. MS is defined as the clustering of abdominal obesity, hypertension, insuline resistance, and dyslipidemia, characterized by low high-density lipoprotein cholesterol (HDLc) and high triglycerides (TG). Hypertriglyceridemia and low HDLc are frequently associated with small dense-low-density lipoprotein (LDLc); as a consequence, preponderance of small dense-LDLc was described in MS [4].

LDL particles are a heterogeneous mixture of lipoproteins differing in density, size, lipid composition, electrical charge, and pathologic properties [5,6]. Determination of LDL particle size is interesting, because the small-dense LDLc can be highly atherogenic due to its ability to penetrate the arterial wall, a low affinity for the receptor, thereby increasing its half-life plasma and low resistance to oxidative stress. It has been described that oxidative modifications of lipoproteins leading to oxidized LDLc, results in biological effects that may contribute to the process of atherosclerosis [7].

Two distinct phenotypes were described: pattern B with a predominance of small, dense LDL particles, and pattern A with a higher proportion of large, more buoyant LDL particles [8]. Although LDL particle size is genetically determined [9], its phenotypic expression may also be affected by environmental factors such as drugs, diet, obesity or exercise. This trait has been called "atherogenic dyslipidemia" and appears to be highly heritable [8]. Factors that shift the LDL subfractions profile towards larger particles may reduce the risk of heart disease [10]. However, several studies have shown that LDL particle size phenotype of risk is not associated with risk fatal myocardial infarction [11].

In order to make a proper evaluation a proper evaluation of lipid-related risk, lipids ratios such as cholesterol/HDLc ratio, TG/HDLc ratio, Apo-B/Apo-AI ratio and AIP (atherogenic index of plasma [Log (TG/HDLC-C)]) should be considered as proposed in several major guidelines [12].

The aim of this study was to establish the importance of LDL particle size measurement in a group of patients with MS attending at a Cardiovascular Risk Unit in Primary Care, by gender and their classification into phenotypes.

## 2. Results

### 2.1. Subjects characteristics

Table 1 shows biochemical parameters and lipid profile by separately by gender and SM status. The results are expressed as mean ± standard deviation, and median and interquartile range.

**Table 1:** Characteristics of the study, by gender and MS status (N = 185)

Variable	Without MS				With MS				*p-*value^3^
	Mean ± SD			*p*-value^1^	Mean ± SD			*p-*value^2^	
	N = 72	Men (N = 37)	Women (N = 35)		N = 113	Men (N = 56)	Women (N = 56)		
**Age (years)**	52.36 ± 18.9	53.8 ± 15.4	50.9 ± 22.3	**0.608**	61 ± 13.8	59.1 ± 13.3	62.9 ± 14.2	**0.068**	0.002*
**Weight (Kg)**	75 ± 12.7	80.9 ± 11.2	68.9 ± 11.3	> 0.001*	86.1 ± 16.3	91.7 ± 12.04	80.5 ± 18.1	> 0.001*	> 0.001*
**Waist circumference (cm)**	94.4 ± 11.1	98.4 ± 8.8	90.3 ± 11.9	0.001*	106.4 ± 11.4	109.1 ± 7.3	103.8 ± 13.9	0.016*	> 0.001*
**SBP (mm Hg)**	128.6 ± 14.9	131.5 ± 13.1	125.4 ± 16.3	**0.073**	144.4 ± 15.9	143.6 ± 15.8	145.3 ± 16.1	**0.369**	> 0.001*
**DBP (mm Hg)**	78 ± 9.7	81.7 ± 9.4	74.1 ± 8.4	0.001*	84.1 ± 8.9	84.4 ± 8.9	83.9 ± 8.9	**0.629**	> 0.001*
**Glucose (mg/dL)**	99 [84.3-147]	106 [91-154.5]	87 [80-144]	0.024*	130.5 [105.3-172]	131 [104.5-163]	130 [106-185]	**0.995**	> 0.001*
**Cholesterol (mg/dL)**	189.6 ± 37.1	189.3 ± 41.6	189.9 ± 32.4	**0.830**	200.3 ± 40.7	194.7 ± 41.9	205.9 ± 39.1	**0.083**	**0.118**
**TG (mg/dL)**	103 [70.8-132]	113 [71.5-141]	84 [68-121]	**0.061**	149 [95.3-212]	156 [101.5-243]	128.5 [91-187]	**0.080**	> 0.001*
**LDL-c (mg/dL)**	107.2 ± 32.4	109.5 ± 35.2	104.7 ± 29.5	**0.787**	113.3 ± 33.6	112.1 ± 36.4	114.5 ± 30.9	**0.459**	**0.274**
**HDL-c (mg/dL)**	59 [52-69]	107 [82-131]	66 [57-74]	> 0.001*	52 [40-61]	45.5 [37-55.8]	56 [49-72]	> 0.001*	> 0.001*
**Apo A-I (mg/dL)**	160.8 ± 21.7	155.4 ± 20.4	166.6 ± 21.8	0.039*	159.2 ± 30.6	147.9 ± 27.2	170.4 ± 29.9	> 0.001*	**0.494**
**Apo A-II (mg/dL)**	31 [28.5-34]	31 [28.3-34.8]	31 [28.5-32]	**0.712**	32 [27.8-39]	32 [27-39]	32.5 [28-39]	**1.000**	**0.119**
**Apo B (mg/dL)**	96.7 ± 24	100.9 ± 25.5	92.12 ± 21.7	**0.147**	108.6 ± 24.5	108.4 ± 22.6	108.9 ± 26.5	**0.806**	0.008*
**Lipoprotein (a) (mg/dL)**	12.7 [4.3-42.9]	15.9 [3.1-39.6]	12.7 [6.2-44.9]	**0.516**	16 [5.3-36.1]	13.1 [4.6-28.3]	19.2 [6.7-48.5]	**0.258**	**0.845**
**LDL particle size (Å)**	273.5 [271-274.7]	271.9 [270.1-274]	274.2 [273-275.4]	0.001*	269 [264.2-272.6]	267.9 [260.6-272]	270.3 [266-274]	0.039*	> 0.001*

SBP: Systolic blood pressure; DBP: Diastolic blood pressure; TG: Triglycerides; LDL-c: dense-low density lipoprotein; HDL-c: high-density lipoprotein.

*p*-value^1^: significance between male and female in non-MS patients.

*p*-value^2^: significance between male and female in MS patients.

*p*-value^3^: significance between non-MS and MS patients.

Notes: Data are mean ± SD, and median and interquartile range. * Statistically significant differences.

According to anthropometric parameters, values (mean values) of weight and waist circumference were significantly higher (*p *> 0.05) in males than in females in those patients without associated MS, as well as levels of blood glucose.

Regarding to classic lipid profile, there only were significant differences by gender in levels of HDLc and Apo A-I. Significantly higher values (*p *> 0.05) were observed in LDL particle size in women compared with men in the group without MS. The results obtained are: HDLc 59 [52-69] mg/dL (107 [82-131] in men and 66 [57-74] mg/dL in women); Apo A-I 160.8 ± 21.7 mg/dL (155.4 ± 20.4 in men and 166.6 ± 21.8 mg/dL in women); and LDL particle size 273.5 [271-274.7] Å (271.9 [270.1-274] in men and 274.2 [273-275.4] Å in women). There were no significant differences in the other parameters.

In MS group, we observed values of HDLc: 52 [40-61] mg/dL (45.5 [37-55.8] in men and 56 [49-72] mg/dL in women) and Apo AI: 159.2 ± 30.6 mg/dL (147.9 ± 27.2 in men and 170.4 ± 29.9 mg/dL in women); they were significantly higher (*p *> 0.05) in women and anthropometric parameters of weight and waist circumference have been found significantly different too. We also found significant differences (*p *> 0.05) by sex in LDL particle size, higher values in women (270.3 [266-273.6] Å) than in men (267.9 [260.6-272] Å).

**Figure 1:**
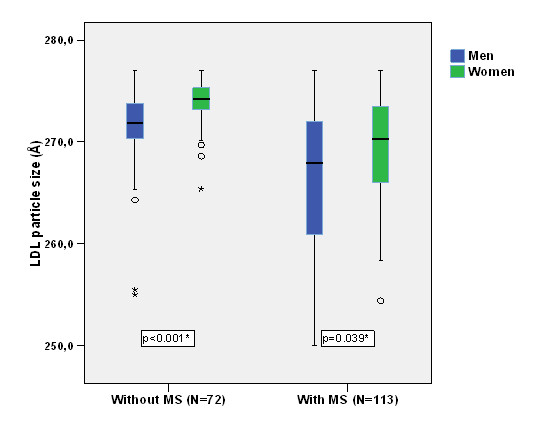
**Distribution of LDL particle size in our population by gender and MS status**.

Distribution of LDL particle size in our population by gender and MS status is shown in Figure 1.

### 2.2. Lipid profile and atherogenic rates

Results of Pearson's correlation analysis between LDL particle size (men and women together) and the other variables (lipid profile and atherogenic rates) are shown in Table 2.

**Table 2:** Pearson's coefficient of LDL particle size with lipid profile and atherogenic rates in our population

LDL particle size	Without MS (N = 72)		With MS (N = 113)	
	r	*p*-value	r	*p*-value
**Plasma cholesterol (mg/dL)**	-0.38	0.001*	-0.02	**0.859**
**Plasma triglycerides (mg/dL)**	-0.69	> 0.001*	-0.19	0.041*
**LDL cholesterol (mg/dL)**	-0.31	0.008*	0.07	**0.472**
**HDL cholesterol (mg/dL)**	0.28	0.019*	0.05	**0.631**
**Apo A-I (mg/dL)**	0.03	**0.833**	0.14	**0.173**
**Apo A-II (mg/dL)**	-0.22	**0.071**	0.01	**0.893**
**Apo B (mg/dL)**	-0.40	0.001*	-0.38	> 0.001*
**Lipoprotein (a) (mg/dL)**	0.07	**0.561**	0.02	**0.851**
**Apo B/Apo A-I ratio**	-0.37	0.002*	-0.37	> 0.001*
**Plasma cholesterol/HDL-c ratio**	-0.50	> 0.001*	-0.09	**0.343**
**Plasma triglycerides/HDL-c ratio**	-0.67	> 0.001*	-0.18	**0.068**
**Log (TG/HDL-c) ratio**	-0.59	> 0.001*	-0.18	**0.056**

* Statistically significant differences.

LDL particle size was significantly (*p *> 0.05) negatively correlated with total cholesterol (r = -0.38, *p *= 0.001), triglycerides (r = -0.69, *p *> 0.001), LDLc (r = -0.31, *p *= 0.008), HDLc (r = 0.28, *p *= 0.019) and Apo-B (r = -0.40, *p *= 0.001) in the group without SM. All atherogenic rates (p > 0.001) were negatively correlated with LDL particle size in this group. We have not found any correlation between LDL particle size and Apo A-I, Apo A-II or Lipoprotein (a).

In SM group, we only found significant differences between particle size and triglycerides (r = -0.19, *p *= 0.041), Apo-B (r = -0.38, *p *> 0.001) and ApoB/ApoAI ratio (r = -0.37, *p *> 0.001), negatively correlated. We have not found any correlation in the rest of lipid profile parameters.

### 2.3. Particle size phenotypes

Table 3 shows lipid profile (concentrations and ratios) by particle size phenotype and SM status.

**Table 3:** Lipid profile: concentrations and ratios, by particle size phenotype and MS status (N = 185)

	Phenotype A (> 260 Å) (N = 127)			Phenotype B (≤ 260 Å) (N = 58)			*p*-value^3^
	No MS	MS	*p*-value^1^	No MS	MS	*p*-value^2^	
**Plasma cholesterol (mg/dL)**	188 ± 35.9	196.1 ± 83.6	**0.279**	245 ± 56	227 ± 48	**0.721**	0.001*
**Plasma triglycerides (mg/dL)**	101 [70-129.3]	129 [91-195]	> 0.001*	282 [265-298]	245 [178-296]	**0.529**	> 0.001*
**LDL cholesterol (mg/dL)**	106.4 ± 31.8	112 ± 34	**0.413**	137 ± 54	124 ± 32	**1.000**	**0.064**
**HDL cholesterol (mg/dL)**	60 [52-69]	53 [41-63]	0.001*	52 [47-56]	42 [38-52]	**0.441**	0.004*
**Apo A-I (mg/dL)**	160.6 ± 22.1	159.37 ± 31.7	**0.632**	168 ± 4.24	158.7 ± 26.5	**0.400**	**0.328**
**Apo A-II (mg/dL)**	31 [28-33]	32 [27-38]	**0.212**	36.5 [35-38]	34 [30-41]	**0.857**	**0.337**
**Apo B (mg/dL)**	95.4 ± 23.1	105.1 ± 21.4	0.031*	139 ± 24.1	122.5 ± 31.2	**0.400**	0.001*
**Lipoprotein (a) (mg/dL)**	18.6 [4.9-43]	14.3 [4.2-33.7]	**0.405**	1.25 [1.2-1.3]	19.5 [12.9-47.3]	0.012*	**0.353**
**Apo B/Apo A-I ratio**	0.60 ± 0.16	0.69 ± 0.19	0.024*	0.83 ± 0.12	0.78 ± 0.18	**0.610**	0.001*
**Plasma cholesterol/HDL-c ratio**	3.22 ± 0.84	3.84 ± 1.18	0.001*	4.72 ± 0.50	4.82 ± 1.17	**0.889**	> 0.001*
**Plasma triglycerides/HDL-c ratio**	1.65 [1.13-2.45]	2.49 [1.53-3.67]	> 0.001*	5.55 [4.73-6.34]	5.49 [3.99-6.85]	**0.963**	> 0.001*
**Log (TG/HDL-c) ratio**	0.20 ± 0.24	0.38 ± 0.29	> 0.001*	0.74 ± 0.08	0.72 ± 0.19	**0.963**	> 0.001*
**LDL particle size (Å)**	273.6 [271.1-274.7]	271.2 [267.7-273.7]	> 0.001*	255.25 [255-255.5]	259 [257.6-260]	**0.114**	> 0.001*

Notes: Data are mean ± SD, and median and interquartile range. * Statistically significant differences.

*p*-value^1^: significance between MS and non-MS in phenotype A patients.

*p*-value^2^: significance between MS and non-MS in phenotype B patients.

*p*-value^3^: significance between phenotypes.

In the non-risk phenotype group (LDL particle size > 260Å), we found significant differences between the groups with and without MS, in triglycerides levels (*p *> 0.001), HDLc (*p *= 0.001), and Apo-B (*p *= 0.031). In addition, values of atherogenic rates: Apo B/Apo A-I ratio (*p *= 0.024), plasma cholesterol/HDLc ratio (*p *= 0.001), plasma triglycerides/HDLc ratio (*p *> 0.001), log (TG/HDLc) ratio (*p *> 0.001), were significantly higher in patients with MS. LDL particle size was significantly lower (*p *> 0.001) in MS patients, and all classic parameters of lipid profile of this group were higher in these group.

When there was phenotype B (LDL particle size ≤ 260Å), we only found significantly higher (*p *= 0.012) values of Lipoprotein (a), when group without MS was compared to patients with MS. However, values of lipid profile parameters such as cholesterol, triglycerides, LDLc, HDLc, Apo A-I and Apo-B were slightly higher in the group without MS. There were no significantly differences (*p *= 0.114) in LDL particle size between patients whitout and with MS when there was risk phenotype.

Lipid profile by SM status and particle size phenotype is shown in Table 4.

**Table 4:** Lipid profile: concentrations and ratios, by MS status and particle size phenotype (N = 185)

	No SM (N = 72)			SM (N = 113)		
	Phenotype A (> 260 Å)	Phenotype B (≤ 260 Å)	*p*-value	Phenotype A (> 260 Å)	Phenotype B (≤ 260 Å)	*p*-value
**Plasma cholesterol (mg/dL)**	188 ± 35.9	245 ± 56	**0.103**	196.1 ± 83.6	227 ± 48	0.009*
**Plasma triglycerides (mg/dL)**	101 [70-129.3]	282 [265-298]	0.003*	129 [91-195]	245 [178-296]	> 0.001*
**LDL cholesterol (mg/dL)**	106.4 ± 31.8	137 ± 54	**0.379**	112 ± 34	124 ± 32	**0.151**
**HDL cholesterol (mg/dL)**	60 [52-69]	52 [47-56]	**0.329**	53 [41-63]	42 [38-52]	**0.072**
**Apo A-I (mg/dL)**	160.6 ± 22.1	168 ± 4.24	**0.538**	159.37 ± 31.7	158.7 ± 26.5	**0.270**
**Apo A-II (mg/dL)**	31 [28-33]	36.5 [35-38]	**0.069**	32 [27-38]	34 [30-41]	**0.934**
**Apo B (mg/dL)**	95.4 ± 23.1	139 ± 24.1	0.025*	105.1 ± 21.4	122.5 ± 31.2	0.014*
**Lipoprotein (a) (mg/dL)**	18.6 [4.9-43]	1.25 [1.2-1.3]	0.005*	14.3 [4.2-33.7]	19.5 [12.9-47.3]	**0.844**
**Apo B/Apo A-I ratio**	0.60 ± 0.16	0.83 ± 0.12	0.050*	0.69 ± 0.19	0.78 ± 0.18	**0.023**
**Plasma cholesterol/HDL-c ratio**	3.22 ± 0.84	4.72 ± 0.50	0.016*	3.84 ± 1.18	4.82 ± 1.17	**0.001**
**Plasma triglycerides/HDL-c ratio**	1.65 [1.13-2.45]	5.55 [4.73-6.34]	0.003*	2.49 [1.53-3.67]	5.49 [3.99-6.85]	> 0.001*
**Log (TG/HDL-c) ratio**	0.20 ± 0.24	0.74 ± 0.08	0.003*	0.38 ± 0.29	0.72 ± 0.19	> 0.001*
**LDL particle size (Å)**	273.6 [271.1-274.7]	255.25 [255-255.5]	0.001*	271.2 [267.7-273.7]	259 [257.6-260]	> 0.001*

Notes: Data are mean ± SD, and median and interquartile range. * Statistically significant differences.

*p*-value^1^: significance between phenotype MS in non-MS patients.

*p*-value^2^: significance between phenotype MS in MS patients.

*p*-value^3^: significance between MS status.

In non-MS group, significant differences were found between particle size phenotypes and lipid parameters: TG (*p *= 0.003), Apo-B (*p *= 0.025), Lipoprotein (a) (*p *= 0.005), and all atherogenic rates (*p *> 0.05).

In MS group, we only founded significant differences: TG (*p *> 0.001), Apo-B (*p *= 0.014) and two atherogenic rates (*p *> 0.001). We found significant differences in LDL particle size in both study groups (*p *= 0.001 and *p *> 0.001 respectively).

## 3. Subjects and methods

### 3.1. Study participants

One hundred eighty-five patients, 93 men (54 ± 14.3 years) and 92 women (58.3 ± 18.6 years), from several areas in South of Spain for a period of one year in a health centre participated in this study. Those patients were attending at a Cardiovascular Risk Unit.

We used a control group of 35 patients (38.5 ± 14.1 years) without any cardiovascular risk factors such as hypertension, diabetes mellitus type 2 or MS. Patients with terminal, kidney, liver disease or thyroid dysfunction were excluded from the study.

Each participant underwent a physical examination, personal interview, collection of biological specimens, and other diagnostic tests. Permission was granted by each community to conduct the study; written informed consent was obtained from all participants.

### 3.2. Physical and metabolic measurements

Anthropometry parameters (weight, waist circumference) were performed with participants fasting, according to standard procedures. Abdominal obesity was defined using Adult Treatment Panel III (ATP III) criteria [13]. Samples of whole blood, plasma and serum were collected from each participant and stored at -80°C.

### 3.3. Definitions of metabolic syndrome

MS was defined according to ATP III criteria [13] as meeting three or more of the following criteria: waist circumference 102 cm for men, 88 cm for women; triglycerides ≥ 150 mg/dL (0.59 mmol/L); HDLc > 40 mg/dL (1.30 mmol/L) for men, > 50 mg/dL (1.04 mmol/L) for women; arterial hypertension (systolic blood pressure ≥ 130 mmHg, diastolic blood pressure ≥ mmHg); fasting glucose ≥ 100 mg/dL (5.56 mmol/L).

### 3.4. Laboratory measurements

Total cholesterol, HDLc and triglycerides were measured by an analyzer Hitachi Modular P (Roche Diagnostics^®^) using a homogeneous enzymatic colorimetric test.

LDLc was estimated using the originally formula described by Friedewald, Levy and Fredrickson [14]. A limitation of this estimate is not to be used when triglycerides are greater than 200 mg/dL.

Determination of apo A-I, apo A-II, apo-B and lipoprotein (a) were performed on an immunonephelometer analyzer (BN Prospec, Siemens Diagnostics^®^) using mono- and polyclonal antibodies. We have considered several atherogenic rates: cholesterol/HDLc ratio, TG/HDLc ratio, Apo-B/Apo-AI ratio and AIP (atherogenic index of plasma [Log (TG/HDLC-C)]). These rates have been calculated from each parameter of classic lipid profile.

### 3.5. Lipoprotein subfraction profile

LDLc subfractions were analyzed by Lipoprint Quantimetrix System^® ^(Quantimetrix Corporation), which uses gel electrophoresis tube with 3% polyacrylamide, following the manufacturer's instructions and procedures described [15]. This system separates the various lipoprotein subfractions according to their different size and electrical charge. It allows isolate up to 12 lipoprotein fractions, and system provides information about the average diameter of LDLc particles, estimated by the algorithm developed by Kazumi et al. [15]. This method has been recently evaluated by our working group [16].

According to the LDLc electrophoretic profile, two phenotypes can be defined: phenotype A with normal total cholesterol mass of the LDLc subfractions, and phenotype non-A (phenotype B) where total cholesterol mass of the LDLc subfractions is intermediate-low.

The average particle size reported by Lipoprint profile is the weighted average (calculated from the area under the curve for each subfraction) of the particle sizes of all the LDLc peaks present in the sample. Based on this size cut-off works out to be above or equal to 260 nm for phenotype A (normal size) and less than for non-A (we call this B).

### 3.6. Statistical analyses

The normal distribution of variables was tested using the Kolmogorov-Smirnov test. Quantitative variables are summarized as mean (m) and standard deviation (SD) and confidence interval 95% (CI), for those who followed a normal distribution and as median and interquartile range otherwise.

To compare means was applied Student t test for normally distributed variables, Mann-Whitney *U*-test for skewed variables.

In addition, the correlation between quantitative variables was assessed by linear regression according to Pearson or Spearman skewed or normally distributed variables. Results were considered significant with *p*-values > 0.05. All statistics were analyzed by using SPSS for Windows v15.0 (Chicago, Illinois, USA).

## 4. Discussion

Predominance of small, dense low-density lipoprotein cholesterol (LDLc) has been designated as an emerging cardiovascular risk factor by the National Cholesterol Education Program Adult Treatment Panel III [3]. MS is a disease with an impact on our society, resulting in large cardiovascular problems. We have considered the importance of evaluating LDL particle size in a population with MS.

In our observational study, we found differences by gender in anthropometric measures (weight and waist circumference), blood pressure and glucose between patients with and without MS. This is in agreement with *Sohn et al*. [17], who found significant differences by gender when compared the association between MS and health-related quality of life of Korean population. In addition, lipid profile was different in all parameters in our two study groups, and gender differences in these parameters within each group were also remarkable in HDLc and Apo A-I values.

When we studied lipid profile (cholesterol, TG, LDLc, HDLc, Apo A-I, Apo A-II, Apo-B, and Lipoprotein (a)) by gender in those patients without and with MS, only significant differences in HDLc and Apo A-I were found. Differences we have found by gender and MS status in LDL particle size are consistent with other authors [18], who showed that males had smaller LDL particle size than females; they studied the presence of small dense LDLc depending on gender and metabolic syndrome, and they also found that patients with MS had LDL particle sizes smaller than those without this syndrome.

Using Pearson's coefficient of LDL particle size with lipid profile and atherogenic rates, we observed inverse relationship between LDL particle size with levels of triglycerides in patients with and without MS, in agreement with the results of *Austin *[19] and *Griffin et al*.[20]; they showed that predominance of small, dense LDLc was associated with an increased risk of coronary artery disease (CAD). Although another study showed that LDL particle size is rarely a significant and independent predictor of CAD risk [21] in patients without MS. We have also observed an inverse relationship between LDL particle size and all atherogenic rates in non-MS patients, likes *Yoshida et al*. [22], who showed that LDLc/Apo B, total cholesterol/TG and LDLc/TG ratios could be used to predict the presence of small dense, but the superiority of these ratios over plasma TG levels alone was not established. In a recent study [23], results indicated a consistent degree of discordance between LDL particle size and lipid indices, as shown our data in SM patients, in which we only found signicantly differences between LDL particle size and Apo B/Apo A-I ratio.

In the study of phenotypes of LDL particle size by SM status, we established two classes of phenotypes based on LDL particle size according to studies of *Griffin *[20] and *Austin and Krauss *[8]; they set the cut-off at 255 Å, while *Rizzo and Berneis *[24] used 258 Å. Other authors [25], classified LDL particle size according to a value of 260 Å; establishing a non-risk phenotype A (> 260 Å), another risk B (≤ 255 Å), and a third intermediate phenotype I (255-260 Å). We have classified study population with a cut-off of 260 Å, grouping phenotype B and intermediate, separating group A (no risk). In our study, only 15% of total population (with and without MS) had phenotype B; however, *Austin et al*. [26] suggested that about 30% of the population could be defined as having the phenotype B, whereas 70% of the population could be classified into phenotype A or into intermediate phenotype.

Into these phenotypes, in non-risk group we found differences in plasma triglycerides, HDLc, Apo-B levels and all atherogenic rates. According to Apo B/Apo A-I ratio, we found significantly differences in non-risk phenotype. *Talmud et al*.[27] showed that Apo B/Apo A-I ratio was associated with the strongest effects on relative risk of coronary heart disease, although some authors showed great discordance compared with either Apo-B or Apo-B/Apo-AI ratio [28].

When we compared patients with and without MS, significant differences were only found in risk phenotype B in lipoprotein (a) levels; we believe that it could be explained by the small sample size (n = 18). Interrelations of two lipoprotein (a) constituents still remain to be elucidated. It is assumed that, once synthesized, apo(a) would associate with LDLc particles currently available in plasma, regardless of their size and density. According to *Nakajima et al*. [29], composition of lipoprotein (a) in an individual would depend on the combination of a genetically controlled apo(a) isoform and a predominant circulating LDLc subclass.

Surprisingly, LDL particle size was smaller in phenotype B patients without MS; this may be due to intrinsic patient factors such as age, gender, dyslipemia or even to other cardiovascular risk factors. We could think that phenotype B 'per se' could be considered as a cardiovascular risk factor.

By MS status, TG, Lp(a) levels and all atherogenic rates were significantly different in non-MS patients and worse in risk phenotype. In MS patients, cholesterol, TG, Apo-B levels and two atherogenic rates were also significantly different.

In conclusion, our data point to LDL particle size is higher in women than in men and in patients without MS. Respect to lipid profile, we also found differences by gender and between groups of patients with and without MS, but these differences were most evident by MS status than by gender.

LDL particle size was inversely correlated mainly with TG and Apo-B levels in presence and absence of MS, although particle size was also negatively significantly correlated with all atherogenic rates in patients without MS.

Phenotype B lipid profile and LDL particle size was worse in non-MS patients. Patients with MS and phenotype B had worse lipid profile and lower LDL particle size, but only significant differences were found TG, Apo B and those atherogenic rates TG related.

We think that LDL particle size could be a worthy marker of risk by itself. Further studies in patient groups with several risk factors will make it possible to establish the importance of particle size of LDL, compared to classic lipid profile in suffered cardiovascular events.

## List of abbreviations

CVD: Cardiovascular Disease; PAD: Peripheral Arterial Disease; MS: Metabolic Syndrome; HDLc: High-density Lipoprotein cholesterol; LDLc: Dense-low density Lipoprotein cholesterol; TG: Triglycerides; Apo-B: Apolipoprotein B; Apo A-I: Apolipopretein A-I; Apo A-II: Apolipoprotein A-II; a: Lipoprotein; AIP: (Lp(a)) Atherogenic Index of Plasma; ATP III: Adult Treatment Panel III; CI: Confidence Interval.

## Authors' contributions

NSR carried out biochemical analysis, performed the statistical analysis and created the manuscript. FVAP carried out biochemical analysis, participated in the design of the study and performed the statistical analysis. EGF carried out the biochemical analysis and participated in the design of the study. AMHM participated in the design of this study. MDAO partipated in the design of this study. PMH carried out participated in the design of the study and its coordination. SPP conceived the study and participated in its design and coordination. All the authors read and approved the final manuscript. The authors declare that they have no competing interests.
